# A diffusible signal factor of the intestine dictates *Salmonella* invasion through its direct control of the virulence activator HilD

**DOI:** 10.1371/journal.ppat.1009357

**Published:** 2021-02-22

**Authors:** Rimi Chowdhury, Paulina D. Pavinski Bitar, Ivan Keresztes, Anthony M. Condo, Craig Altier

**Affiliations:** 1 Department of Population Medicine and Diagnostic Sciences, College of Veterinary Medicine, Cornell University, Ithaca, New York, United States of America; 2 Chemistry NMR Facility, Cornell University, Ithaca, New York, United States of America; University of California Davis School of Medicine, UNITED STATES

## Abstract

Successful intestinal infection by *Salmonella* requires optimized invasion of the gut epithelium, a function that is energetically costly. *Salmonella* have therefore evolved to intricately regulate the expression of their virulence determinants by utilizing specific environmental cues. Here we show that a powerful repressor of *Salmonella* invasion, a *cis-*2 unsaturated long chain fatty acid, is present in the murine large intestine. Originally identified in *Xylella fastidiosa* as a diffusible signal factor for quorum sensing, this fatty acid directly interacts with HilD, the master transcriptional regulator of *Salmonella*, and prevents *hilA* activation, thus inhibiting *Salmonella* invasion. We further identify the fatty acid binding region of HilD and show it to be selective and biased in favour of signal factors with a *cis*-2 unsaturation over other intestinal fatty acids. Single mutation of specific HilD amino acids to alanine prevented fatty acid binding, thereby alleviating their repressive effect on invasion. Together, these results highlight an exceedingly sensitive mechanism used by *Salmonella* to colonize its host by detecting and exploiting specific molecules present within the complex intestinal environment.

## Author summary

Survival of pathogens in the complex intestinal environment depends upon their ability to sense their surroundings. For successful infection, these pathogens have evolved to recognize and utilize specific environmental cues to regulate virulence at appropriate niches. *Salmonella* employs diverse spatial cues in the host intestine to optimize expression of its invasion machinery at the appropriate location. Here we show that a *cis*-2 unsaturated diffusible signal factor, *cis*-2-hexadecenoic acid, known to be a potent repressor of *Salmonella* invasion, is present in the murine large intestine. We elucidate how this quorum sensing long chain fatty acid can directly bind to the virulence master regulator HilD and prevent intestinal invasion, thus providing insight into how *Salmonella* controls invasion at appropriate locations in the host intestine.

## Introduction

Enteric pathogens employ sophisticated strategies to sense their environment and accordingly express virulence factors to colonize their mammalian hosts [1–5]. Most Gram-negative enteric pathogens rely on an elaborate virulence apparatus for infection, whose expression and assembly costs the bacterium in growth rate and fitness [6]. Thus, enteric pathogens have evolved to induce virulence exclusively in response to specific environmental cues in the intestinal tract [2]. *Salmonella*, a Gram-negative enteric bacteria and one of the leading causes of gastro-intestinal tract infections worldwide, similarly recognizes specific cues in the intestinal environment that dictate its behaviour in that niche [7–9].

To invade the intestinal epithelium, a function essential to both carriage and disease, *Salmonella* uses a macromolecular syringe known as the Type 3 Secretion System 1 (T3SS-1) [10]. The construction of this machinery, encoded within *Salmonella* Pathogenicity Island 1 (SPI-1), involves activation of about forty genes, whose expression is controlled by the AraC-type transcriptional regulators HilD, HilC and RtsA [11,12]. These regulators activate transcription of *hilA*, which initiates expression of structural proteins of the T3SS-1 [13,14]. Each of these regulators can also induce expression of *hilD*, *hilC*, and *rtsA*, in addition to *hilA*, forming an intricate feed-forward regulatory loop to activate SPI-1 expression [15]. Most of the regulatory signals that govern SPI-1 expression control this system primarily through HilD [7,16–20]. Thus HilD is known as the leading controller of this cascade, while HilC and RtsA function to strengthen the system [11,15,17,21–23].

Several environmental signals are known to target this cascade to control SPI-1 activation [7–9,24,25]. These include short- and long chain fatty acids (LCFAs) produced in the intestine as by-products of dietary lipid digestion or metabolic remnants of the resident microbiota. Acetate, primarily produced by cellulose breakdown [26], promotes invasion by increasing translation of the *hilD* mRNA [17,27,28]. Formate, present primarily in the ileum, has been shown to induce invasion in a *hilD* dependent manner [8]. In contrast, propionate and butyrate, present in high concentrations in the caecum and colon, repress invasion gene expression [9,25,27]. Similarly oleic acid, a LCFA copiously present in bile, has been shown to repress invasion gene expression by directly binding to HilD, thereby preventing it from binding to promoter DNA and activating downstream gene transcription [7]. It has long been speculated that such repressive LCFAs, present in specific locations of the intestine, can act as signals that dictate *Salmonella* invasion.

Several Gram-negative bacteria employ a unique class of fatty acids to detect cell density in their environments. These quorum-sensing molecules have been implicated in intra-species, inter-species and inter-kingdom communication, where they regulate virulence, motility, biofilm formation, antibiotic tolerance and persistence [29–35]. These compounds are LCFAs with a *cis*-unsaturation at the second carbon and are known as Diffusible Signal Factors (DSFs). DSFs are produced through the cronotase RpfF that has both enoyl-CoA dehydratase and thioesterase activities and acts on the acyl-acyl carrier protein (acyl-ACP), an intermediate produced during the fatty acid synthesis pathway [29]. RpfF introduces the hallmark *cis*-unsaturation at the second carbon by its dehydratase activity, and cleaves ACP by its thioesterase activity to release a *cis*-2 unsaturated free fatty acid. These fatty acids are exported into the extracellular milieu for perception by other bacteria. RpfF of plant pathogen *Xyllela fastidiosa* produces the DSF *cis*-2-hexadecenoic acid (c2-HDA) that has been shown to drastically reduce invasion gene expression in *Salmonella* by reducing the half-life of HilD and preventing it from binding to the *hilC* promoter [36].

Here we show that the DSF c2-HDA is present in the murine large intestine. We further show that this DSF directly binds to HilD with high affinity, resulting in the loss of HilD DNA binding activity, thus preventing transcription of *hilA* and activation of downstream invasion genes. Mutation of key residues of HilD, predicted to interact with the DSF, ameliorated the repressive effect of c2-HDA on *Salmonella* invasion. Together, these results identify a novel signalling molecule within the intestine that is perceived by *Salmonella* to dictate its invasive behaviour.

## Results

### The invasion-repressing signal factor c2-HDA is present in the murine intestine

**Fig 1:**
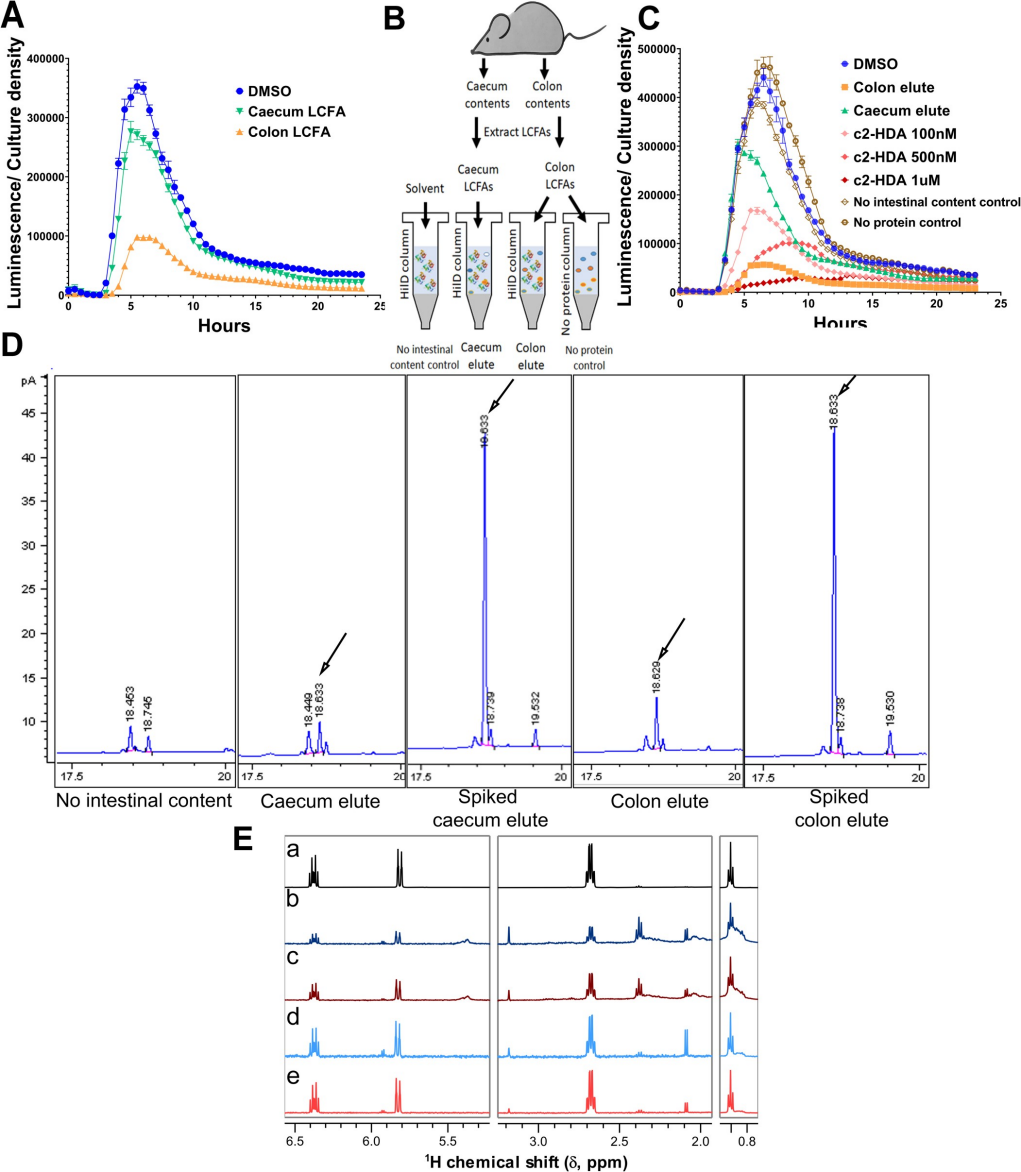
The invasion-repressing signal factor c2-HDA is present in the murine intestine. (A) *Salmonella* strain carrying *hilA-luxCDABE* transcriptional reporter fusion was grown in presence of LCFAs extracted from murine caecum or colon; luminescence was normalized to bacterial culture density. Data show mean ±SD of five replicates. (B) Summary of the HilD-affinity experiments. Caecal and colon contents of C57BL/6 mice (n = 15) were pooled, LCFAs were extracted and applied to HilD-His bound to TALON beads. Columns were washed and HilD-fatty acid complex was eluted. Control columns where no extract was applied (no intestinal content) and columns without HilD (no protein control) were treated similarly. All elutes underwent organic extraction and were finally dissolved in DMSO. (C) *Salmonella* strain carrying *hilA-luxCDABE* transcriptional reporter fusion was grown in the presence of caecal or colon elutes from HilD affinity columns or with c2-HDA. Luminescence was normalized to bacterial culture density. Data show mean ±SD of five replicates. (D) GC spectra of caecal and colon elutes from HilD affinity columns, and their c2-HDA (40 μM) spiked counterparts. c2-HDA characteristic peak at 18.6 minutes are marked with arrow. (E) ^1^H-NMR spectra of c2-HDA standard (a), LCFAs extracted from caecum (b) and colon (c) of C57BL/6 mice (n = 16) and their respective elutes from HilD affinity columns (d-e).

LCFAs harboring a *cis-*oriented unsaturation at their second carbon strongly inhibit *Salmonella* invasion by preventing the binding of the invasion activator HilD to its DNA targets and inducing its rapid degradation [36]. As molecules of this class are produced by the Gammaproteobacteria and employed in quorum sensing, we sought to determine whether they exist as chemical signals within the mammalian intestine, and thus constitute a *bona fide* means by which *Salmonella* invasion might be modulated. We therefore extracted LCFAs from the large intestinal contents of mice and tested their ability to repress invasion genes. Using a *luxCDABE* reporter fusion to *hilA*, an essential SPI-1 invasion regulator under the control of HilD, we found that LCFAs extracted from the murine colon reduced *hilA* gene expression when added to growing cultures of *Salmonella* by 3.6-fold, while those from the caecum reduced significantly, but less effectively, by 22% (**Fig 1A**). To identify the specific fatty acids present in the intestine capable of binding HilD, we next used a protein affinity strategy (**Fig 1B**). We applied LCFAs extracted from the colon and caecum to resin-bound His-tagged HilD, washed and eluted the protein, and then extracted the fatty acids that had bound to HilD. Fatty acids eluted from HilD that had been treated with colonic LCFAs greatly repressed *hilA* expression by 7.7-fold (**Fig 1C**). The repression was equivalent to that of 0.5–1 μM c2-HDA, the most potent inhibitor of this class identified previously. Fatty acids eluted from HilD treated with caecal LCFAs also repressed *hilA* expression considerably but less potently (31%), being less effective than the equivalent of 50 nM c2-HDA. To identify these fatty acids, we next analyzed them by gas chromatography (GC). We found several compounds not observed in the sham extracts used as controls (**S1 Table**). Prominent among these was a compound with a retention time of 18.6 minutes, identical to that of c2-HDA (**Fig 1D**). Addition of commercial c2-HDA to the samples produced a single, more prominent peak at 18.6 minutes, suggesting identity with c2-HDA. ^1^H-NMR analysis of these samples confirmed the presence of prominent downfield-shifted vinylic resonances characteristic of *cis*-2 unsaturated LCFA at 6.37 ppm (dt, *J* = 11.75, 7.6 Hz) and 5.83 ppm (dt, *J* = 11.5, 1.8 Hz) (**Figs 1E** and **S3**). These findings thus demonstrate that a potent chemical inhibitor of *Salmonella* invasion that is known to be synthesized by bacteria and that functions by binding and inhibiting HilD exists in the murine intestine in sufficient concentrations to repress invasion gene expression.

### Specific amino acid residues of HilD are essential for repression by c2-HDA

**Fig 2:**
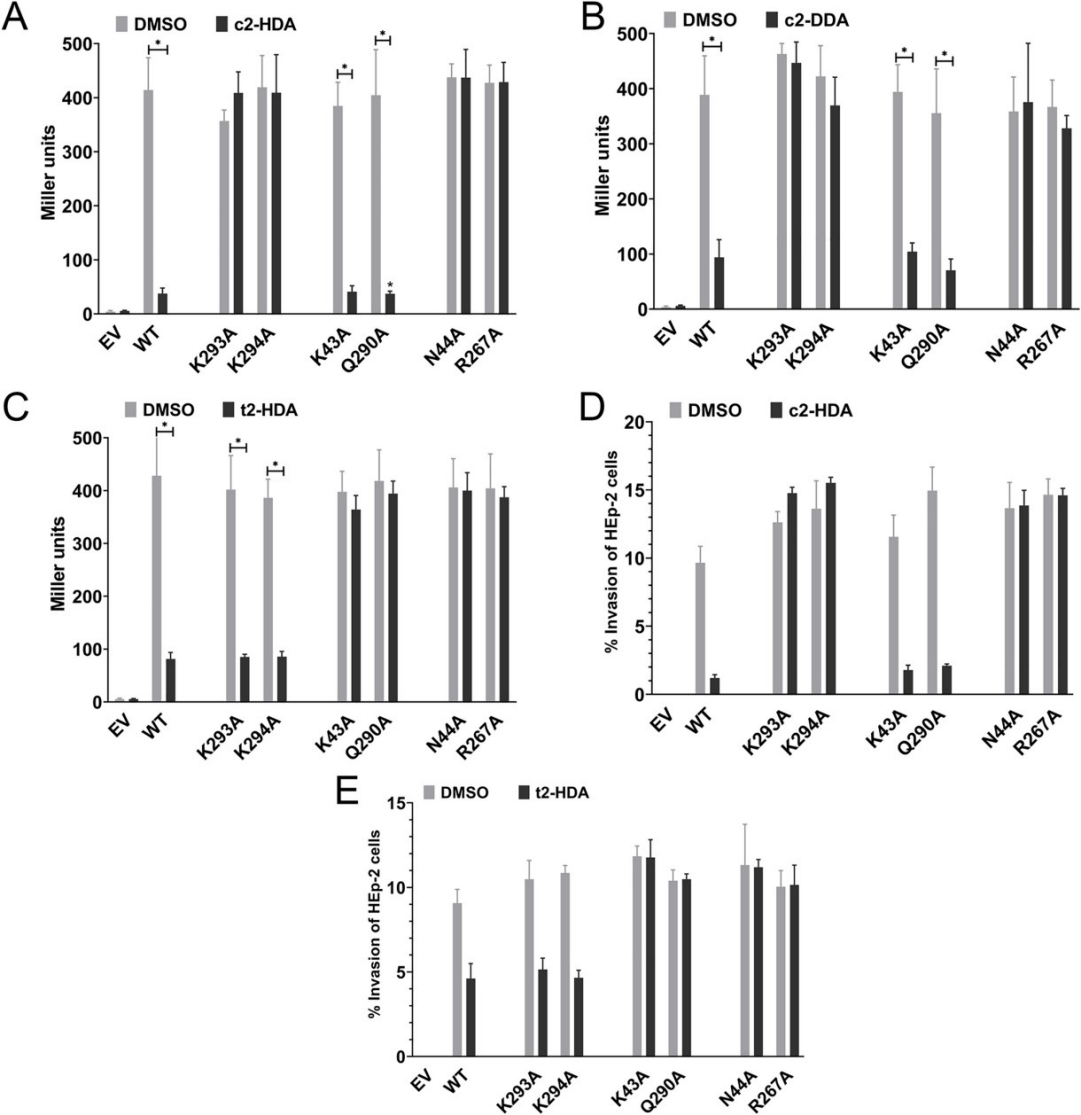
Specific amino acid residues of HilD are essential for repression by c2-HDA. (A-C) *Salmonella* strains expressing empty vector (EV) or wild type (WT) or mutant HilD were grown in the presence of 20 μM c2-HDA (A), c2-DDA (B), or t2-HDA (C) and expression of the invasion gene *sipB* was measured using a *lacZY* transcriptional reporter fusion, by β-galactosidase assays. Bars represent mean ±SD (n = 4). Differences between respective DMSO controls and fatty acid treatments were calculated by Mann-Whitney test, * p<0.05. (D-E) Strains were grown in the presence of 20 μM c2-HDA (D) or t2-HDA (E) and invasion of HEp-2 cells was measured by gentamicin protection assay. Data show mean ±SD (n = 4).

To investigate how c2-HDA interacts with HilD, we used *in silico* methods to visualize their interaction. In the absence of an established structure of HilD, we used the X-ray crystal of its structural and functional homolog, ToxT of *Vibrio cholerae*, to create a virtual HilD replica by homology modeling (**S1A Fig**). We virtually docked c2-HDA onto this HilD model and found eight amino acid residues (T41, N44, T51, K129, F134, R267, K293 and K294) whose side-chains were predicted to be in close proximity to the ligand (**S1B Fig**). To test these predictions, we replaced each identified HilD residue with an alanine, expressing the mutant constructs on a low copy-number plasmid. To isolate the effects of the *hilD* mutations from other SPI-1 activators, we introduced these constructs into a *Salmonella* strain with null mutations in chromosomal *hilC*, *rtsA*, and *hilD*, thus eliminating all components of the invasion feed-forward regulatory loop. Using *sipB*::*lacZY*, a representative HilD-regulated gene, as a reporter, we examined SPI-1 gene expression in cultures grown with 20 μM c2-HDA. All of the *hilD* mutants retained their full capacity to induce *sipB* in the absence of c2-HDA, complementing the chromosomal *hilD* null mutant, and demonstrating that the point mutations did not reduce transcriptional activation by HilD (**Fig 2A**). We found HilD mutants K293A and K294A to be resistant to the repressive effects of c2-HDA. No significant repression of *sipB* was observed in these mutants, compared to almost 11-fold reduction in the strain with a wild type HilD. Interestingly, the effect of disrupting residues K293 and K294 on invasion gene expression was specific only to LCFAs with a *cis*-2 unsaturation. The LCFA *cis*-2-dodecenoic acid (c2-DDA), which reduced *sipB* expression by 4.1-fold in the wild type HilD strain, produced no significant reduction in the K293A and K294A mutants (**Fig 2B**). Conversely, *trans*-2-hexadecenoic acid (t2-HDA), which differs from c2-HDA only in the spatial orientation of unsaturation at the second carbon, repressed *sipB* expression in mutant K293A (4.7-fold) and mutant K294A (4.5-fold) as well as wild type HilD (5.2-fold) (**Fig 2C**). Similarly, LCFAs with a saturated second carbon and a *cis*-unsaturation at another position, such as *cis*-8-eicosenoic acid (c8-EA) and oleic acid (*cis*-9-octadecenoic acid), continued to repress *sipB* expression in mutants K293A and K294 (**S1C** and **S1D Fig**). These results thus identify the amino acids of HilD important for invasion gene repression by the *cis*-2 class of fatty acids.

To investigate how HilD interacts with LCFAs without a *cis*-2 unsaturation, we used our *in silico* model to identify amino acid residues in proximity to the ligand when presented with oleic acid, a fatty acid that is a known constituent of the intestine but does not contain a *cis*-2 unsaturation. *In silico* docking showed that residues K43 and Q290 had now replaced residues K293 and K294 in proximity to the LCFA (**S1E Fig)**. Testing of alanine-replacement mutants showed that while oleic acid reduced *sipB* expression by 3.7-fold in wild type HilD, it no longer had a repressive effect on HilD mutants K43A and Q290A (**S1D Fig**). Repression by c2-HDA, however, remained unaffected in both mutants (**Fig 2A**). Similar patterns of repression were observed with t2-HDA (**Fig 2C**), indicating that LCFAs without a *cis*-2 unsaturation repress invasion genes through HilD residues K43 and Q290.

Of the other HilD residues identified by *in silico* docking, residues N44 and R267 were consistently found to be adjacent to the carboxylic acid group, common to all fatty acids. In accordance with our predictions, alanine replacements of these residues made *sipB* refractory to repression by all LCFAs (**Fig 2A–2C**). While *sipB* expression in wild type HilD was repressed significantly by every LCFA tested, no significant repression was found in HilD mutant N44A or R267A by any tested LCFA, confirming our prediction. These effects on invasion-gene expression were further manifested in the invasive ability of *Salmonella*. HEp-2 cell monolayers were infected with *Salmonella* grown overnight in the presence of different LCFAs, and cell invasion was assessed by a gentamicin protection assay. As predicted, *Salmonella* expressing HilD^K293A^ invaded HEp-2 cells despite the presence of 20 μM c2-HDA, a concentration that greatly inhibited invasion of *Salmonella* expressing wild type HilD (8-fold) (**Fig 2D**). The identical concentration of its *trans*-isomer t2-HDA prevented invasion by *Salmonella* expressing the wild type HilD or HilD^K293A^ (each 2-fold), but had no effect on *Salmonella* expressing HilDQ^290A^ (**Fig 2E**). Invasion by a strain expressing HilD^N44A^ was unaffected by either c2-HDA or t2-HDA (**Fig 2D–2E**). Taken together, these results indicate that different classes of LCFAs require distinct residues of HilD for repression defined by subtle differences in the structures of the LCFAs.

### c2-HDA outcompetes other LCFAs for repression of invasion

**Fig 3:**
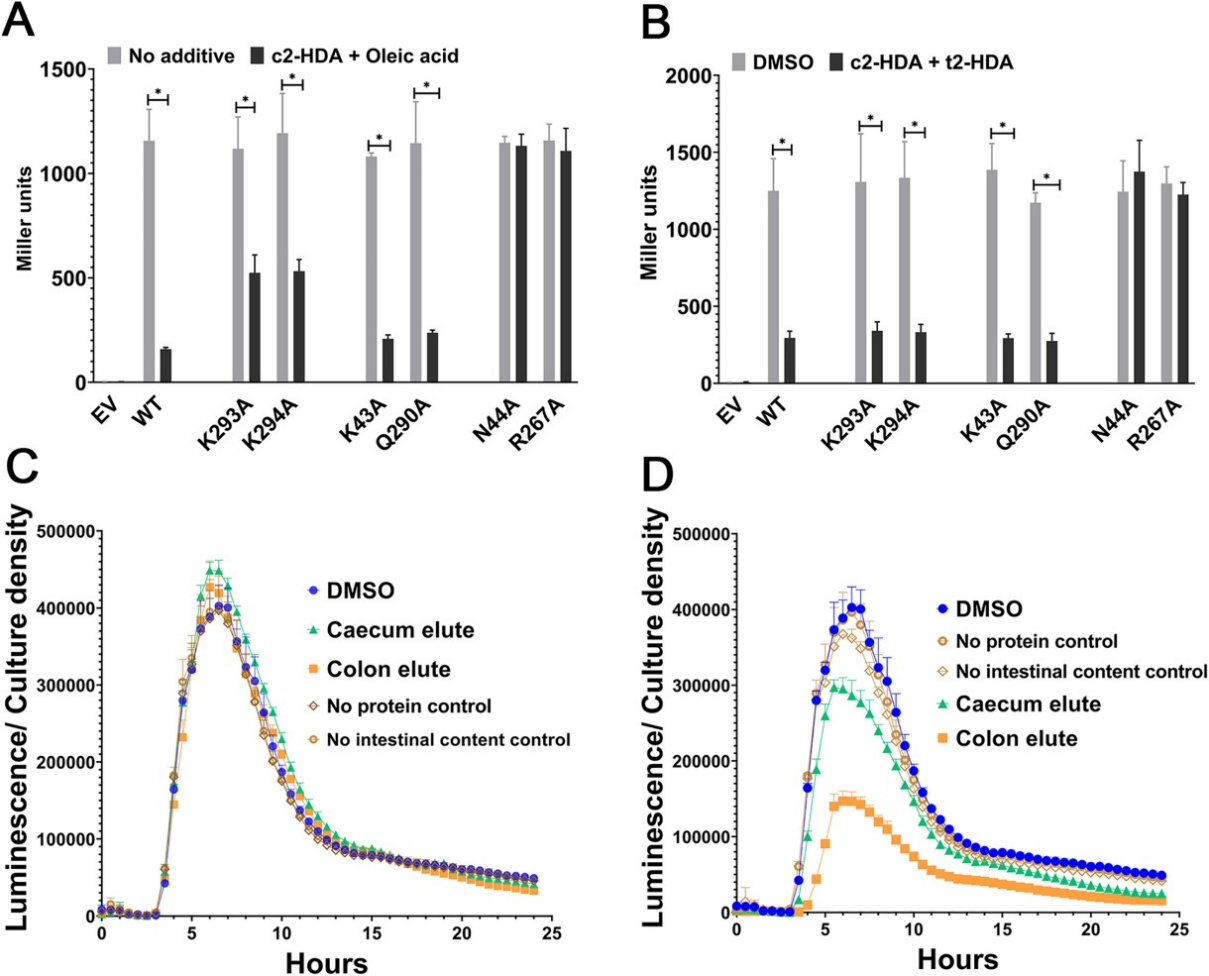
c2-HDA outcompetes other repressive LCFAs. (A-B) *Salmonella* strains expressing empty vector (EV) or wildtype (WT) or mutant HilD were grown in the presence of 20 μM of each of c2-HDA, and oleic acid (A) or t2-HDA (B) and expression of *sipB*::*lacZY* was measured by β-galactosidase assays. Bars represent mean ±SD (n = 4). Differences between respective untreated/ DMSO controls and fatty acid treatments were calculated by Mann-Whitney test, * p<0.05. (C-D) *Salmonella* strain carrying *hilA-luxCDABE* transcriptional reporter fusion was grown in the presence of caecal or colon elutes from HilD^K293A^ (C) or HilD^Q290A^ (D) affinity columns. Luminescence was normalized to bacterial culture density. Data show mean ±SD of five replicates.

The intestine is a complex environment, known to contain a variety of fatty acids, yet our HilD affinity assay selectively captured c2-HDA. The model we have proposed creates the possibility of competition among fatty acids for a single attachment site, with c2-HDA as the preferred ligand. We specifically tested whether such competition exists by growing *Salmonella* strains expressing wild type or mutant HilD in the presence of equal concentrations of c2-HDA and oleic acid, an intestinal LCFA saturated at its second position [7]. *sipB* expression was repressed 10.9-fold by c2-HDA (**Fig 2A**) and 3.7-fold by oleic acid (**S1D Fig**), but 7.2-fold by the mix of the two LCFAs (**Fig 3A**). The lack of an additive effect when comparing LCFA mixtures to their individual components suggests that only one LCFA can mediate repression at a time, implicating the existence of a single, or overlapping, LCFA attachment site in HilD. Additionally, mutants that remained responsive only to c2-HDA (HilD^K43A^ and HilD^Q290A^) had significantly lower levels of *sipB* expression (5.2- and 4.8-fold respectively) than mutants that were responsive only to oleic acid (HilD^K293A^, 2.1-fold and HilD^K294A^, 2.2-fold) when the two LCFAs were provided together. Similar results were obtained for the combination of c2-HDA and t2-HDA (**Fig 3B**) and c2-HDA and twice the concentration of oleic acid (**S1F Fig**), showing that c2-HDA is a more potent repressor, and suggesting that it prevails in competition with other fatty acids for a common attachment site in HilD.

To ensure that this compound was the predominant invasion-repressing LCFA of the large intestine, we repeated the HilD-affinity approach using two mutant proteins: HilD^K293A^ which should trap all LCFAs without a *cis*-2 unsaturation, and HilD^Q290A^, which should trap all LCFAs having a *cis*-2 unsaturation. We found that LCFA extracts of murine caeca and colon affinity-purified using HilD^K293A^ failed to repress *hilA*, while those purified using HilD^Q290A^ retained this repressive capacity (**Fig 3C** and **3D**). These results thus indicate that the predominant signal for invasion repression in the murine large intestine harbors a *cis*-2 unsaturation, likely c2-HDA, and that this molecule preferentially represses invasion through HilD in competition with other intestinal LCFAs.

### c2-HDA binds to HilD with higher affinity than other repressive LCFAs

**Fig 4:**
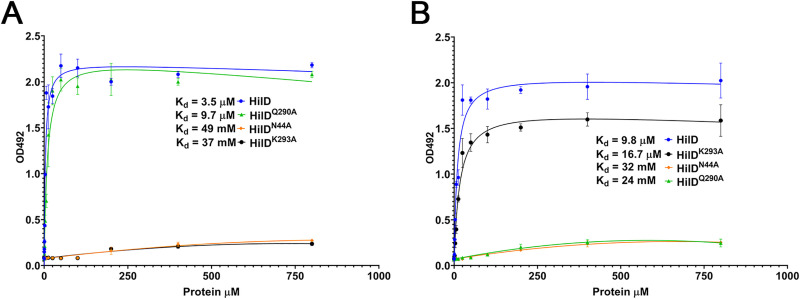
c2-HDA binds to HilD with higher affinity than to other repressive LCFAs. (A-B) Increasing concentrations of wild type HilD or mutant proteins were added to 96-well plates pre-adsorbed with 10 μM c2-HDA (A) or t2-HDA (B). Binding was detected by anti-His antibody. Non-linear regression was plotted to calculate K_d_.

The results presented above strongly suggest direct binding between HilD and various LCFAs. To investigate this, we expressed wild type and mutant HilD proteins and compared their binding affinities to c2-HDA by ELISA. As a control, we used the *trans*-isomer t2-HDA, which differs from c2-HDA only in the spatial orientation of its unsaturation. As expected, we found that wild type HilD protein showed high binding affinity to both c2-HDA (K_d_ = 3.5 μM) and t2-HDA (K_d_ = 9.8 μM) (**Fig 4A** and **4B**). Consistent with our model, HilD^K293A^, which lacks one of the residues required for the repressive effects of LCFAs with a *cis*-2 unsaturation, showed negligible binding to c2-HDA (**Fig 4A**), but bound to the *trans*-isomer t2-HDA efficiently (K_d_ = 16.7 μM) (**Fig 4B**). Similarly, HilD^Q290A^, which our results have demonstrated to be unaffected by LCFAs having a saturated or *trans*-unsaturated second carbon, bound to c2-HDA as efficiently as wild type HilD (K_d_ = 9.7 μM), but failed to bind to the *trans*-isomer. Finally, HilD^N44A^, suspected to interact with the polar carboxylic acid head group of all LCFAs, showed essentially no binding to either fatty acid (**Fig 4A** and **4B**). These results demonstrate the direct binding of LCFAs to HilD, and suggest that the LCFA binding site in HilD consists of residues that interact with a moiety common to all fatty acids, the polar carboxylic acid group, as well as residues that are adaptive and differentially selective to the presence of a *cis*- unsaturation at the second carbon.

### Binding of c2-HDA to specific HilD residues dictates its stability and capacity to bind target DNA

**Fig 5:**
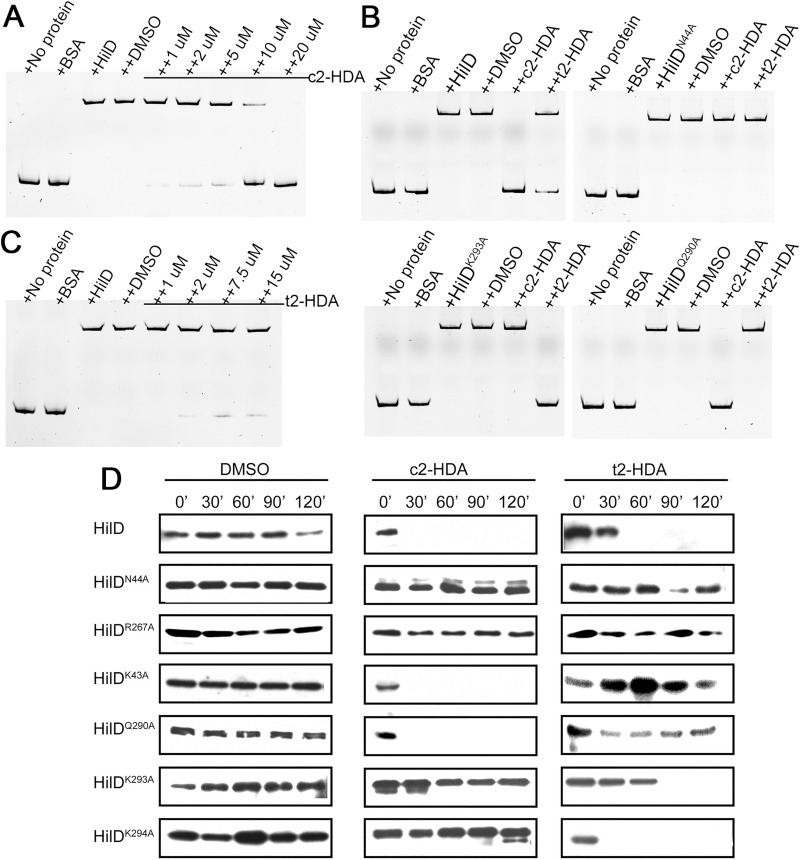
Binding of c2-HDA to specific HilD residues dictates its stability and capacity to bind target DNA. (A,C) EMSA of *hilA* promoter DNA (10 nM) in the presence of 150 μM BSA or wild type HilD with increasing concentrations of c2-HDA (A) or t2-HDA (C). (B) EMSA of *hilA* promoter DNA (10 nM) in the presence of 150 μM BSA or wild type HilD or mutant proteins with 30 μM c2-HDA or t2-HDA. (D) Western blot of HilD degradation assay. *Salmonella* strains carrying wild type or mutant *hilD*-3XFLAG constructs were grown in the presence of 20 μM c2-HDA or t2-HDA. Protein expression was halted and cultures were collected at indicated time points to analyze the stability of HilD by western blotting.

We have demonstrated that c2-HDA binds directly to the transcriptional activator HilD and represses invasion genes. We next sought to determine whether this binding disrupts the ability of HilD to bind its target DNA. To that end, we performed electrophoretic mobility shift assays (EMSAs), incubating wild type or mutant HilD with a *hilA* promoter probe in the presence of differing concentrations of c2-HDA. We found that increasing concentrations of c2-HDA progressively impaired interaction of wild type HilD with the DNA target, thus facilitating its migration through the polyacrylamide gel **(Fig 5A)**. HilD^K293A^, which is impaired for binding to c2-HDA, remained bound to the *hilA* promoter despite the presence of 30 μM c2-HDA **(Fig 5B)**. Similarly, HilD^N44A^, predicted to lack the ability to bind the carboxyl group of LCFAs and impervious to the effects of c2-HDA, also retained its binding of target DNA (**Fig 5B)**. We repeated these assays using varying concentrations of the *trans*-isomer t2-HDA, which, though less potent than c2-HDA, is also capable of repressing invasion through direct binding to HilD. Consistent with our previous results, the *trans*-isomer t2-HDA efficiently reduced the DNA binding capacity of the wild type HilD and HilD^K293A^ (**Fig 5B** and **5C**). However, this LCFA did not disrupt DNA binding by mutants of moieties predicted to interact with the *trans*-unsaturated second carbon, such as HilD^Q290A^, or the polar carboxylic acid head group of LCFAs, such as HilD^N44A^ (**Fig 5B**). Taken together, these results show that binding by different classes of LCFAs to specific residues of HilD reduces its DNA binding ability and thus the induction of invasion.

We have previously shown that LCFAs reduce invasion gene expression by inducing the rapid degradation of HilD by Lon protease [36]. To investigate if the binding of c2-HDA to specific residues of HilD targets the protein for degradation, we measured wild type and mutant HilD levels in *Salmonella* grown in the presence of LCFAs. We constructed *Salmonella* strains carrying each *hilD* construct under the control of a tetracycline-inducible promoter and fused to a C-terminal 3XFLAG tag to quantify HilD levels. These strains were grown in the presence of 20 μM c2-HDA for two hours, protein expression was halted, and stability of existing protein was assessed by immunoblotting. Rapid degradation of HilD was observed when the strain expressing the wild type allele was grown in the presence of c2-HDA **(Fig 5D)**. In contrast, mutant HilD proteins unable to bind to LCFAs with the *cis*-2 bond (HilD^K293A^ and HilD^K294A^) and those unable to bind any LCFA tested (HilD^N44A^ and HilD^R267A^), were protected from degradation. HilD mutants that remained susceptible to LCFAs with a *cis*-2 unsaturation (HilD^K43A^ and HilD^Q290A^) were degraded as rapidly as wild type HilD. We further found that wild type HilD was degraded when grown in the presence of the *trans*-isomer t2-HDA, while HilD mutant proteins unable to bind to LCFAs with the *trans*-unsaturated second carbon (HilD^K43A^ and HilD^Q290A^), and those unable to bind to any LCFA (HilD^N44A^ and HilD^R267A^), escaped degradation. As expected, HilD mutant proteins that remain affected by LCFAs with a *trans*-2-unsaturation (HilD^K293A^ and HilD^K294A^) were rapidly degraded, highlighting the mechanism by which different classes of LCFAs bind and control HilD protein levels and subsequently invasion. These results lay out the precise mechanism by which *Salmonella* invasion regulator HilD uses c2-HDA, a diffusible signal factor of the gut, to control activation of invasion and regulate its behavior in the host intestine.

## Discussion

The intestine is a complex chemical environment shaped by the interaction of the host with the resident microbiota, replete with biochemical signals that pathogens have evolved to recognize to perfect their infection process. As *Salmonella* moves through the intestinal tract, it recognizes many such compounds and uses them as spatial cues; to survive and multiply or to adhere to and invade tissues by expressing its invasion machinery. Here we have identified and described a delicate molecular mechanism used by *Salmonella* to regulate its virulence in the host intestine. Our results show that *Salmonella* perceives a quorum-sensing molecule used by species of the Gammaproteobacteria, the DSF c2-HDA, that is present in the murine caecum and colon. HilD, the master regulator of *Salmonella* invasion, interacts with this signal using residues that recognize both its ubiquitous carboxylate head group and its characteristic *cis*-2 unsaturation. The resulting loss of attachment by this transcriptional regulator of invasion to its target DNA averts activation and expression of downstream SPI-1 genes, thus controlling epithelial invasion.

The gut harbors a multitude of cues that affect *Salmonella* invasion-gene expression [2]. In addition to diet and bile, the metabolic byproducts and cellular remnants of the native microbiota, such as LCFAs, provide a rich source of such inhibitors [7]. The DSF c2-HDA belongs to a rare class of LCFAs produced by a crotonase of Gammaproteobacteria, encoded by *rpfF*, and exported into the extracellular milieu for perception and quorum sensing. Several genera harboring homologs of *rpfF* are a part of the natural microbiota of the gut, including *Burkholderia*, *Xanthomonas* and *Enterobacter* [37]. Further, the DSF-producing bacterial species *Stenotrophomonas maltophilia* was found to be part of the crypt-specific core microbiota of the murine colon [33,38]. This suggests that c2-HDA is likely produced in the gut by resident microbiota as a quorum-sensing molecule and has been co-opted by *Salmonella* for virulence regulation.

To colonize the intestine, invading pathogens, such as *Salmonella*, must compete with the indigenous microbiota [39]. This colonization resistance has been attributed primarily to competition for restricted nutrients between invaders and residents, and to the production of toxic metabolites by the resident microbiota [40]. Studies have shown that removal of indigenous microbiota by streptomycin pretreatment facilitates *Salmonella* invasion of the large intestinal tract and produces significantly higher inflammation and colitis [40,41]. Owing to the invasion repressive strength of DSFs, it may be speculated that the presence of DSF-producing bacteria in the gut contributes to the colonization resistance which may explain the massive inflammation generated by *Salmonella* in germ-free mice or streptomycin-treated mice.

While c2-HDA and other repressive LCFAs may prevent overwhelming infections by *Salmonella*, they may also serve as essential cues used by the pathogen itself to modulate its virulence as a means to promote its survival and proliferation. SPI-1 expression and subsequent invasion of *Salmonella* requires a delicate balance, induced in a minority of the population (~10% of the infecting inoculum), that generates inflammation necessary for survival of the majority of the population [12,42–46]. Hence, such repressive fatty acids, present at specific locations in the gut, may provide a growth advantage to *Salmonella* by restricting SPI-1 expression sufficiently to induce the low level of inflammation needed to promote pathogen growth, while maintaining the competitive fitness of the majority of the population. Such a model predicts that HilD has been tuned through evolution to be optimally affected by these signals. Indeed, the mutations of HilD described here that make it unresponsive to repressive LCFA signals have not been identified in field strains of *Salmonella*, suggesting that control by these signals is important to the fitness of the pathogen. Our findings also suggest a spatial gradient of invasion repression as *Salmonella* traverses the gut: The chemical cues of the ileum, the proximal portion of the gut, favor the induction of invasion [8,27], while those of the large intestine, the distal portion of the gut, repress invasion [9,25,27]. Consistent with this model, we show in this work that repressive LCFA signals are more prevalent in the colon, the most distal portion of the gut, than in the cecum that precedes it. Taken together, these findings indicate that *Salmonella* has evolved to reduce, but not eliminate, invasion as it moves though the gut, as a means to optimize its proliferation and transmission. Essential to such control is the composition of the gut microbiota. c2-HDA is not known to be produced by animals, but instead by a select group of bacteria. It is thus highly likely that specific species of bacteria that produce c2-HDA reside within the large intestine of the mice used in our study. It therefore follows that infection and colonization of *Salmonella* may vary based upon the composition of the microbiota. For example, O’Loughlin et al. showed that *Stenotrophomonas*, known to produce a DSF, is a negligible part of native microbiota of CBA/J mice [47]. The lack of such a species in the native microbiota may thus contribute to the robust inflammation observed in caecum and colon of this mouse strain, and underscores the importance of understanding the interactions of pathogens with the microbiota in experimental animal models.

Our conclusions suggest a model by which HilD interacts with the broad class of LCFAs to the detriment of its DNA-binding ability. Additionally, it suggests a delicate way in which HilD discriminates between the DSF c2-HDA and other LCFAs. Amino acid alignment of the homologous *Vibrio cholerae* ToxT to HilD shows that residue R267 aligns with K230 of ToxT (**S2 Fig**). It has been shown that binding of the polar head group of fatty acids to ToxT K230 and K31 serves to bridge the N- and C-terminal domains of this protein into a “closed” conformation, making it incapable of dimerization and DNA binding [48,49]. In this study we have shown the requirement of HilD residues R267 of the C-terminal domain and N44 of the N-terminal domain for the transcriptional function of HilD (**Fig 2**). It is therefore similarly plausible that the carboxylate head group, integral to all fatty acids, forms close interactions with residues R267 and N44, consequently bringing the N- and C-terminal domains of HilD into close proximity, thus straining and altering the conformation of the DNA-binding domain. A high-resolution crystal structure of HilD will be needed to confirm this hypothesized structural shift.

Finally, we have shown that the fatty acid interacting site of HilD is able to discern and preferentially respond to the DSF c2-HDA over LCFAs without the *cis*-2 conformation. Furthermore, Bosire et al [36] demonstrated that c2-HDA is superior amongst *cis*-2 unsaturated fatty acids of various carbon lengths in its control of SPI-1 gene expression. These findings suggest that the 16-carbon tail with a *cis*-2 unsaturation is the optimal LCFA to be accommodated into the binding pocket in an energetically favorable conformation, which may explain the increased potency of repression by c2-HDA over other *cis*-2 unsaturated LCFAs. Taken together, these findings lead us to propose that *Salmonella* has adapted its generalized fatty acid binding pocket into a sensitive mechanism of spatial perception to stringently control virulence.

## Materials and methods

### Ethics statement

Studies involving vertebrate animals were approved by the Cornell University Institutional Animal Care and Use Committee. Euthanasia was conducted using carbon dioxide inhalation in accordance with the American Veterinary Medical Association Guidelines for Euthanasia of Animals. The Cornell University Animal Care and Use program and associated animal facilities are operated in accordance with the U.S. Department of Agriculture Animal Welfare Act (1966), Regulation (C.F.R., 2009) and policies, the Health Research Extension Act (1985), the Public Health Service Policy on Humane Care and Use of Laboratory Animals (PHS, 2002), the Guide for the Care and Use of Laboratory Animals (NRC, 2011), the Guide for the Care and Use of Agricultural Animals in Research and Teaching (2010), the New York State Health Law (Article 5, Section 504), and other applicable federal, state, and local laws, regulations, policies, and guidelines.

### Cells, strains and fatty acids

All experiments were performed using *Salmonella enterica* subsp. enterica serovar Typhimurium 14028s, or *E*. *coli* BL21 DE3 unless otherwise noted. All strains used are described in Table 1. HEp-2 cells (ATCC: CCL-23) were maintained in Dulbecco’s modified eagle’s medium supplemented with 10% fetal bovine serum. Fatty acid c2-DDA was purchased from Sigma (49619-10MG). c8-EA (76261-96-6), oleic acid (90260) and t2-HDA (11132) were purchased from Cayman Chemicals. c2-HDA was bought from both Cayman chemicals (11133) and Larodan (10–1609).

**Table 1:** Strains and plasmids used in the study.

****Strain****	****Genotype****	****Reference****
CA4787	Δ*rtsA* Δ*hilC sipB*::*lacZY hilD*::cam *recD*::Tn*10*	This study
**Plasmids**		
pBA426	*hilA-luxCDABE*	[36]
pQE60-*hilD*-6XHis	*hilD*-6XHis	This study
pQE60-*hilDN44A*-6XHis	*hilDN44A*-6XHis	This study
pQE60-*hilDQ290A*-6XHis	*hilDQ290A*-6XHis	This study
pQE60-*hilDK293A*-6XHis	*hilDK293A*-6XHis	This study
pET15b(+)	6XHis	Novagen
pET15b(+)-*hilD*	6XHis-*hilD*	This study
pET15b(+)-*hilDN44A*	6XHis-*hilDN44A*	This study
pET15b(+)-*hilDQ290A*	6XHis-*hilDQ290A*	This study
pET15b(+)-*hilDK293A*	6XHis-*hilDK293A*	This study
pWSK29	P_tetRA_-3XFLAG	[12]
pCA211	pWSK29-P_tetRA_*-hilD*-3XFLAG	[12]
	pWSK29-P_tetRA_*-hilDK43A*-3XFLAG	This study
	pWSK29-P_tetRA_ *-hilDR267A*-3XFLAG	This study
	pWSK29-P_tetRA_*-hilDQ290A*-3XFLAG	This study
	pWSK29-P_tetRA_*-hilDK293A*-3XFLAG	This study
	pWSK29-P_tetRA_*-hilDK294A*-3XFLAG	This study
	pWSK29-P_tetRA_*-hilDN44A*-3XFLAG	This study

### Construction of *hilD* mutations

To test the effect of mutations in *hilD*, we initially constructed a background *Salmonella* strain without chromosomal *hilC*, *rtsA* and *hilD* and fused *sipB* to *lacZY* to assess SPI-1 expression [50]. *hilD* with a C-terminal 3XFLAG tag was cloned into pWSK29 under control of the *tetRA* promoter for tetracycline-inducible expression. This plasmid was subjected to site-directed mutagenesis at specific nucleotides to change selected amino acids to alanine, using the Agilent QuikChange II Site-Directed Mutagenesis kit following their protocol. Mutations were confirmed by DNA sequencing.

### Animal experiments

Carcasses of C57BL/6 mice were procured from the Cornell Center for Animal Resources and Education. Caecum and colon were removed and cut longitudinally. Organs were vigorously vortexed in 1 ml PBS to release their contents. Debris was removed by centrifugation and clear supernatants were collected.

### Organic extraction

Samples were mixed with equal volumes of chloroform and vortexed vigorously. The separated organic fraction was collected and the process was repeated three times. Organic fractions from all extractions were pooled and evaporated to remove chloroform. Dried extracts were dissolved in DMSO for assays or derivatized for GC.

### LCFA extraction

Caecal or colonic contents were subjected to Dole’s extraction method [46]. Briefly, samples were mixed with five volumes of extraction mix (Isopropanol: heptane: sulphuric acid::39:10:1) and vortexed, followed by addition of two volumes of water and six volumes of heptane. After vortexing, organic phase was removed and evaporated. Dried samples were dissolved in DMSO for assays, deuterated chloroform (CDCl_3_) for ^1^H-NMR, or PBS with 10% DMSO for protein-affinity studies.

### Protein-affinity columns

Wild type or mutated *hilD* sequences were cloned in pQE60 with C-terminal 6XHis tag. Plasmids were electroporated into *E*. *coli* BL21. Cultures were induced with 5 mM IPTG at 37° C for 4 hours followed by centrifugation. Pellets of 50 ml culture were dissolved in 5 ml equilibration buffer (50 mM sodium phosphate, 300 mM sodium chloride, lysozyme 1 mg/ml, pH 7.4) and incubated on ice for 30 minutes. Triton X-100 and DNAse 1 were added to a final concentration of 1% and 5 μg/ml respectively, and samples were rocked in 4°C for 1 hour. Insoluble cell debris was removed by centrifugation at 13,000 x *g* and the clarified supernatant was applied to columns pre-equilibrated with 250 μl TALON Metal Affinity Resin. Flow-through was discarded and columns were washed three times with Wash buffer (50 mM sodium phosphate, 300 mM sodium chloride, pH 6.5). To confirm presence of HilD or mutant proteins, 10 μl of respective lysate and resin were run on SDS-PAGE. Finally, protein-resin columns were re-suspended in 1 ml of PBS-10% DMSO.

LCFA extracts from caecum or colon in PBS-10% DMSO were applied to respective columns and allowed to bind for 6–8 hours at 4°C. Flow-through was discarded and resin was washed three times with PBS-10% DMSO. Bound protein-fatty acid complex was eluted by applying 500 μl elution buffer (50 mM sodium phosphate, 300 mM sodium chloride, 500 mM Imidazole, pH 5.0). Fatty acids were extracted from the elute by organic extraction. After drying, samples were dissolved in DMSO for expression assays, deuterated chloroform for ^1^H-NMR, or derivatized for GC.

### GC sample preparation

Dried samples were dissolved in 100 μl of derivatization mix (equal volumes of 3-(Trifluoromethyl) phenyltrimethylammonium hydroxide and Hexane) and incubated at room temperature overnight. After centrifugation, 50 μl supernatant was added to GC vials with inserts; 5 μl of each sample was tested (Agilent 6890N). Spectra were analyzed in Chemstation.

### NMR experiments

Dried samples were dissolved in 600 μl of deuterated chloroform and transferred to NMR tubes. NMR experiments were performed at 25° C on a Bruker AVIII HD spectrometer equipped with a broadband Prodigy cryoprobe operating at 499.76 MHz for ^1^H observation. ^1^H spectra were acquired with spectral window from -4 to 16 ppm, 32 scans, 3.3 s acquisition time, 30 s relaxation delay and 90° excitation pulse (12 μs). The data were processed and analyzed using MestReNova (14.1.1, Mestrelab Research). FIDs were zero-filled to 64k data points and 1 Hz Gaussian window function was applied prior to Fourier transform. Manual phase-correction and baseline correction (7^th^-order polynomial) were applied, and the spectra were referenced to residual CHCl_3_ at δ = 7.28 ppm.

### *In silico* studies

Amino acid sequence of HilD was retrieved from NCBI (https://www.ncbi.nlm.nih.gov/ Accession NP_461796.1). Virtual models were generated in Modeller (https://salilab.org/modeller/) using ToxT (PDB: 3GBG) as template. The structure with the lowest Discrete Optimized Protein Energy score was assessed and improved by energy minimization. Quality of the HilD model was further assessed using PROCHECK, ERRAT and VERIFY3D from Structure Analysis and Verification Server (https://servicesn.mbi.ucla.edu/SAVES/). RAMPAGE was used to assess the Ramachandran plot of this model. For docking, structures of fatty acids were retrieved from PubChem (https://pubchem.ncbi.nlm.nih.gov/). Ligands and HilD were prepared for docking by LigPrep and Protein Prep Wizard in Schrodinger Maestro using default parameters. Induced fit docking was performed in Glide. Docked complexes were analyzed in Maestro.

### β-Galactosidase assays

*Salmonella* strains were inoculated into LB buffered with 100 mM MOPS [3-(N-morpholino) propanesulphonic acid] pH 6.7, with necessary antibiotics, and fatty acids or control solvents to be tested. Cultures were incubated at 37° C without aeration overnight, and assays were performed using an established protocol [24].

### Luciferase assays

*Salmonella* strains carrying *luxCDABE* reporter plasmids were grown overnight in LB with the necessary antibiotics, followed by subculture in M9 minimal medium with 0.2% glucose, antibiotics and 1 mM nonanoic acid (to repress background SPI-1 expression) for 16 hours. Final cultures were washed three times with PBS to remove treatments. Bacteria were inoculated at a starting OD_600_ of 0.02 into 150 μL of LB containing 100 mM MOPS pH 6.7, the necessary antibiotics and various treatments. Luminescence was measured every 30 minutes for 24 hours using a Biotek Synergy H1 microplate reader.

### Invasion assay

Invasion was quantified by gentamicin protection assay [36,51,52]. HEp-2 cells were seeded into 24-well cell culture plates (~10^5^/well) and allowed to attach overnight at 37° C with 5% CO_2_). *Salmonella* strains were grown overnight without aeration in LB buffered with 100 mM HEPES, pH 8.0, with antibiotics and fatty acids or control solvent. Overnight cultures were washed with sterile PBS. Bacteria were added to the HEp-2 cell monolayer (MOI 1:10), centrifuged briefly (10 minutes at 200 x *g*) and incubated for 1 hour at 37°C. Wells were washed three times with sterile PBS followed by addition of media with 200 μg/mL gentamicin and incubated for 1 hour. Cells were then washed three times with sterile PBS and lysed by 0.1% Triton X-100. Intracellular bacteria were enumerated by spreading lysates onto LB agar plates with appropriate selection.

### HilD half-life assay

The HilD half-life was assessed as previously described [24]. *Salmonella* strains having plasmids with tetracycline-inducible *hilD* or its mutated sequence with a C-terminal 3XFLAG tag and a *sipB*::*lacZ* fusion (for invasion gene expression assessment) were grown overnight and then diluted 1:100 into LB buffered with 100 mM MOPS (pH 6.7) with appropriate antibiotics and fatty acids or control solvents. After 2 hours of growth, aliquots were collected to quantitate *sipB*::*lacZ* expression in a β-galactosidase assay. Individual cultures were then equilibrated to an OD_600_ of 1.0 to ensure equal density at the half-life assay beginning point. New protein production was halted by addition of a cocktail of antibiotics (rifampin, streptomycin and spectinomycin). These cultures were incubated at 37°C and aliquots were collected every 30 minutes for 2 hours. Samples were lysed in 5X sample buffer, boiled, and immunoblotted with an anti-FLAG antibody to monitor HilD.

### Recombinant protein expression and purification

*hilD* or its mutated sequences were amplified and cloned into pET15b(+) under an N-terminal 6xHis tag. The constructs were transformed into *E*. *coli* BL21 (DE3) for expression. Transformants were induced with 5 mM IPTG for 4 hours. Cultures were centrifuged, lysed and treated with TALON beads as described above. Bound protein was eluted by applying 1 ml elution buffer to the columns. Elution was confirmed by SDS-PAGE. Following confirmation, eluted samples were dialyzed overnight in dialysis buffer (50 mM sodium phosphate, 300 mM NaCl, 10% glycerol). Protein concentration and quality were assessed by Bradford estimation and SDS-PAGE.

### ELISA

96-well polystyrene plates were coated with 10 μM fatty acid in coating buffer (0.05 M sodium carbonate-bicarbonate, pH 9.3) and incubated overnight at 4°C. Wells were washed three times with PBS, followed by addition of blocking buffer (1% Ficoll 400 in PBS). After blocking, wells were washed three times with PBS, and different concentrations of proteins in blocking buffer were added and incubated at room temperature for 2 hours. Wells were washed and incubated with 1:1000 dilution of anti-His tag antibody in blocking buffer at room temperature for an hour. After washing, wells were incubated with 1:5000 dilution of anti-mouse secondary antibody for an hour. Finally, wells were washed three times with PBS-T (0.1%) and OPD substrate was added. After color development, reaction was stopped with 1N sulfuric acid and read spectrophotometrically at 490 nm. Binding was analyzed in GraphPad Prism.

### EMSAs

These were performed as previously described [36]. Briefly, 10 nM *hilA* promoter DNA (-286 to +31) was mixed with 150 nM HilD in a binding buffer (100 mM Tris pH 7.5, 10 mM EDTA, 1M KCl, 1 mM DTT, 50% v/v glycerol). Fatty acids or control solvents were added at various concentrations. Binding reaction was performed at room temperature for 1 hour. Dye solution was added to final concentration of 1X and samples were separated on 10% acrylamide gels. DNA was stained using SYBR Safe and visualized in BioRad GelDoc imaging system.

### Statistical analysis

All experiments were repeated twice and data of representative experiment have been shown. K_d_ was calculated by using Total One-Site binding in GraphPad Prism. Means of treated and control samples were compared in GraphPad Prism using Mann-Whitney test. Differences were considered significant when p<0.05 and indicated by *.

## Supporting information

S1 Table GC peak list. List of total peaks obtained from GC of caecal and colon elutes from HilD affinity columns. Bold represents unique peaks. c2-HDA peaks at 18.6 minutes have been highlighted.
(DOCX)

S1 Fig Amino acid residues of HilD required for repression by LCFAs. (S1A) Predicted HilD model showing N- and C-terminus. (S1B) Predicted binding site of c2-HDA in HilD. In c2-HDA, C atoms are green, O atoms are red and alpha carbon is marked as C^1^. (S1C and S1D) *Salmonella* strains expressing empty vector (EV) or wild type (WT) or mutant HilD were grown in presence of 20 μM c8-EA (S1C) or Oleic acid (S1D) and expression of the invasion gene *sipB* was measured using a *lacZY* transcriptional reporter fusion, by β-galactosidase assays. Bars represent mean ±SD (n = 4). Differences between respective controls and fatty acid treatments were calculated by Mann-Whitney test, * p<0.05. (S1E) Predicted binding site of oleic acid in HilD. In oleic acid, C atoms are green, O atoms are red and alpha carbon is marked as C^1^. (S1F) *Salmonella* strains expressing empty vector (EV) or wildtype (WT) or mutant HilD were grown in the presence of 10 μM of c2-HDA and 20 μM of oleic acid and expression of *sipB*::*lacZY* was measured by β-galactosidase assays. Bars represent mean ±SD (n = 4). Differences between respective untreated/ DMSO controls and fatty acid treatments were calculated by Mann-Whitney test, * p<0.05.
(TIF)

S2 Fig Alignment of HilD amino acid sequence with ToxT. Amino acid sequences were aligned using PROMALS3D. R267 of HilD and K230 of ToxT are marked.
(TIF)

S3 Fig Full spectra of ^1^H-NMR. Full display (left) and expansion (right) of 500 MHz ^1^H-NMR spectrum of c2-HDA (A1 and A2), LCFAs extracted from caecum (B1 and B2) and colon (D1 and D2) and their respective elutes from HilD affinity columns (C1 and C2, E1 and E2) of C57BL/6 mice (n = 16) in CDCl_3_ at 25° C.
(TIF)
