# Supplementary figures and images for: A diffusible signal factor of the intestine dictates Salmonella invasion through its direct control of the virulence activator HilD
Source: PLoS Pathog. 2021 Feb 22;17(2):e1009357. doi: 10.1371/journal.ppat.1009357 (PMC7932555; doi:10.1371/journal.ppat.1009357)

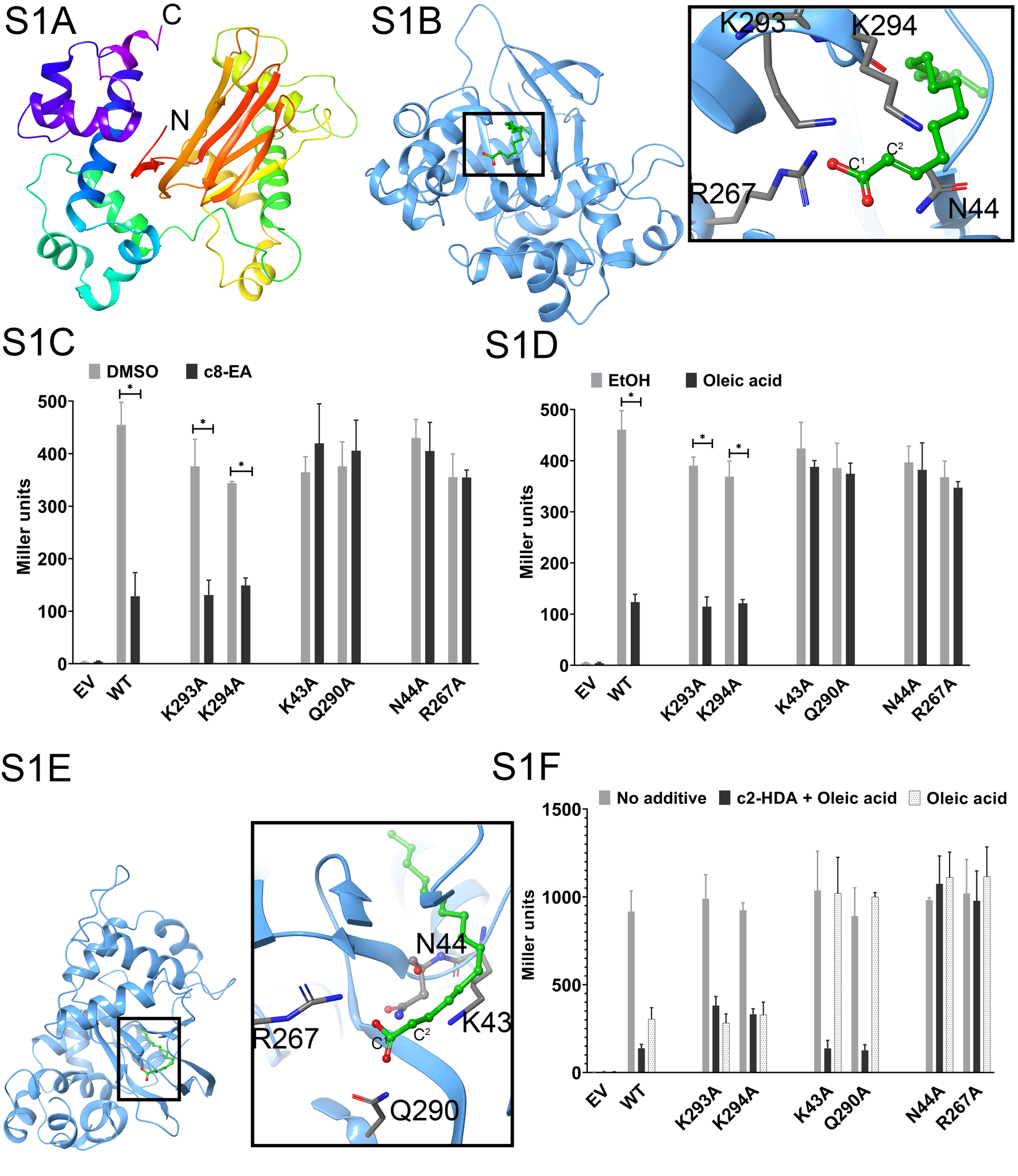

Supplement: S1 Fig — (S1A) Predicted HilD model showing N- and C-terminus. (S1B) Predicted binding site of c2-HDA in HilD. In c2-HDA, C atoms are green, O atoms are red and alpha carbon is marked as C1. (S1C and S1D) Salmonella strains expressing empty vector (EV) or wild type (WT) or mutant HilD were grown in presence of 20 μM c8-EA (S1C) or Oleic acid (S1D) and expression of the invasion gene sipB was measured using a lacZY transcriptional reporter fusion, by β-galactosidase assays. Bars represent mean ±SD (n = 4). Differences between respective controls and fatty acid treatments were calculated by Mann-Whitney test, * p<0.05. (S1E) Predicted binding site of oleic acid in HilD. In oleic acid, C atoms are green, O atoms are red and alpha carbon is marked as C1. (S1F) Salmonella strains expressing empty vector (EV) or wildtype (WT) or mutant HilD were grown in the presence of 10 μM of c2-HDA and 20 μM of oleic acid and expression of sipB::lacZY was measured by β-galactosidase assays. Bars represent mean ±SD (n = 4). Differences between respective untreated/ DMSO controls and fatty acid treatments were calculated by Mann-Whitney test, * p<0.05. (TIF) [file ppat.1009357.s002.tif]

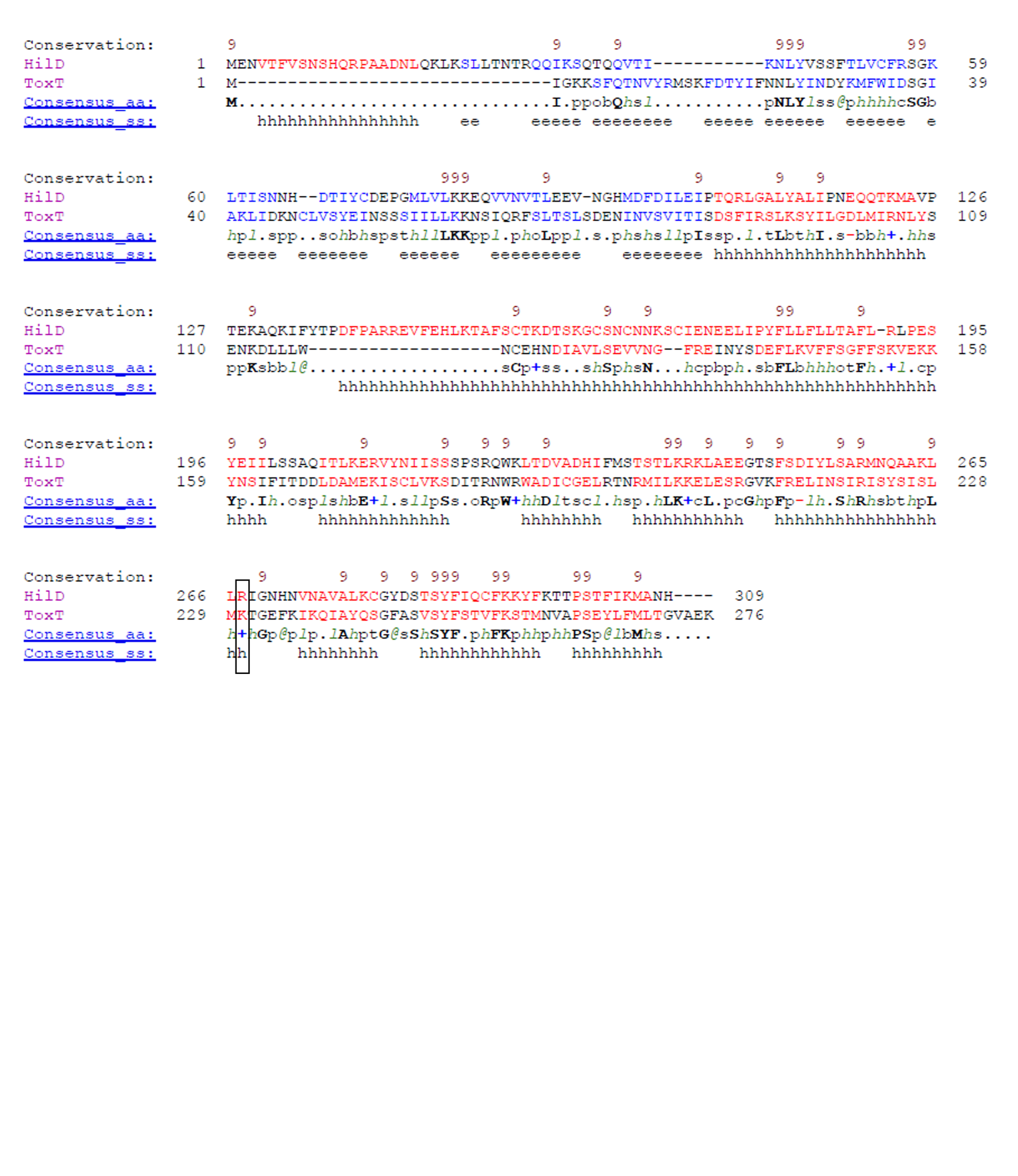

Supplement: S2 Fig — Amino acid sequences were aligned using PROMALS3D. R267 of HilD and K230 of ToxT are marked. (TIF) [file ppat.1009357.s003.tif]

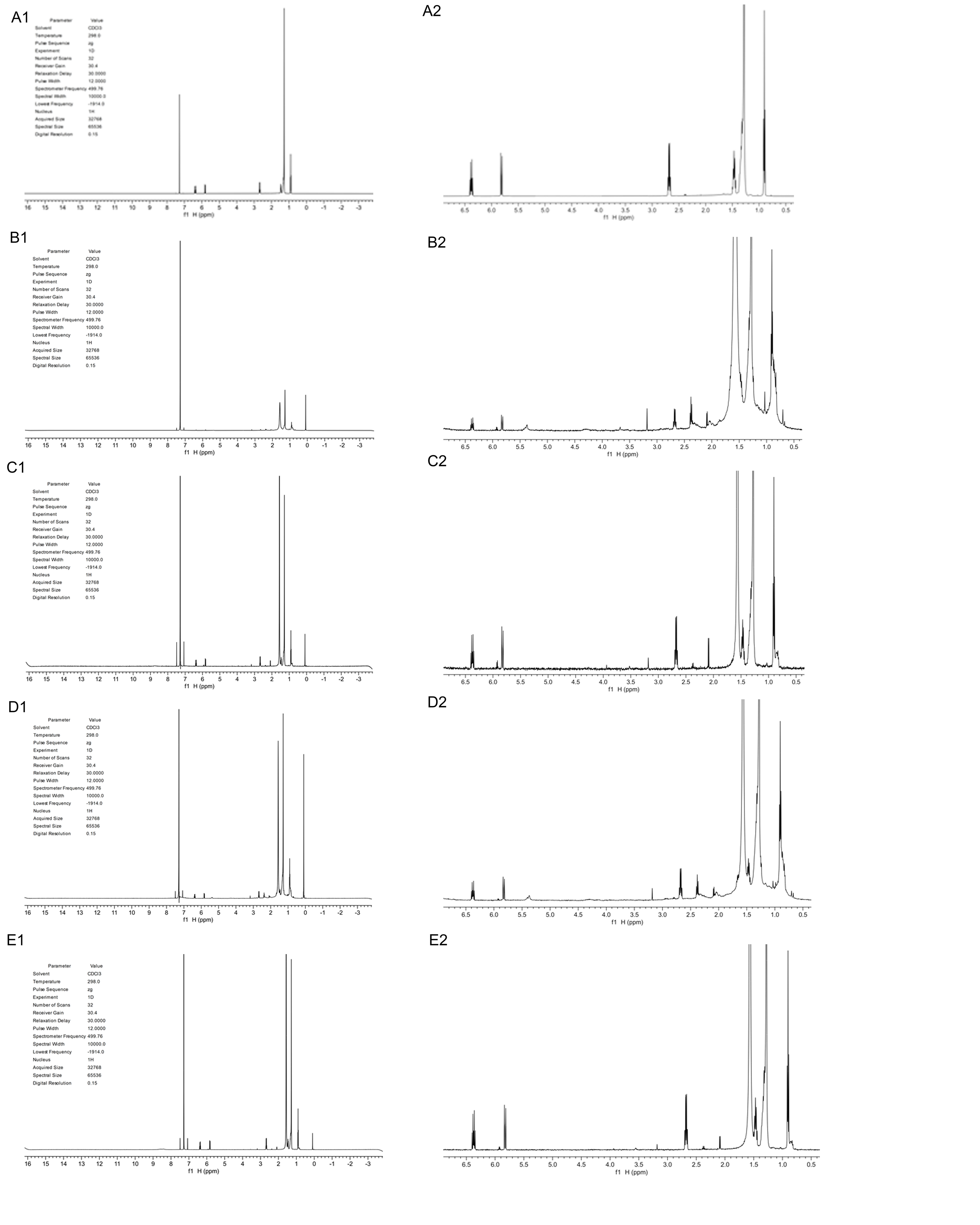

Supplement: S3 Fig — Full display (left) and expansion (right) of 500 MHz 1H-NMR spectrum of c2-HDA (A1 and A2), LCFAs extracted from caecum (B1 and B2) and colon (D1 and D2) and their respective elutes from HilD affinity columns (C1 and C2, E1 and E2) of C57BL/6 mice (n = 16) in CDCl3 at 25° C. (TIF) [file ppat.1009357.s004.tif]
